# Molecular characterization of antibiotic-resistant *Staphylococcus aureus* from livestock (bovine and swine)

**DOI:** 10.14202/vetworld.2017.598-604

**Published:** 2017-06-05

**Authors:** Asima Zehra, Randhir Singh, Simranpreet Kaur, J. P. S. Gill

**Affiliations:** School of Public Health and Zoonoses, Guru Angad Dev Veterinary and Animal Sciences University, Ludhiana, Punjab, India

**Keywords:** antibiotic resistance genes, epsilometer test, livestock nasal swabs, multidrug resistance, *Staphylococcus aureus*

## Abstract

**Aim:**

The aim of this study was to figure the prevalence, phenotypic and genotypic antibiotic resistance (AR) pattern of *Staphylococcus aureus* isolated from bovine and swine nares.

**Materials and Methods:**

Colonies with typical morphology on Baird-Parker agar supplemented with egg-yolk tellurite emulsion were selected and biochemically/genotypically identified as *S. aureus*. These strains were further subjected to epsilometer test for their sensitivity to various clinically important antibiotics and antibiotic susceptibility testing for amoxicillin/clavulanic acid, and double-disk diffusion testing was performed by the standard disc diffusion method following CLSI guidelines. *S. aureus* strains were also tested for the presence of AR genes, viz., *bla*Z, *mec*A, *aac*A-aphD, *erm* (*erm*A, *erm*B, *erm*C), tet (efflux genes *tet*K and *tet*L, *tet*M and *tet*O of the ribosomal protection family), and *van*A.

**Results:**

The nasal cavities of 17 out of 47 randomly selected bovine and 20 out of 28 randomly selected swine were positive for *S. aureus*, representing the prevalence of 36.2% (95% confidence interval [CI]: 22.5-49.9) and 71.4% (95% CI: 54.7-88.1), respectively. Most of the *S. aureus* strains showed higher resistance to penicillin (94.6%, minimal inhibitory concentration [MIC] ≥1.5 µg/ml) followed by ciprofloxacin (56.7%, MIC ≥32 µg/ml) and tetracycline (18.9%, MIC ≥32 µg/ml). About 10-15% of the strains were resistant to gentamicin (MIC 16 µg/ml) and oxacillin (MIC 6-8 µg/ml). None of the strains were resistant to vancomycin (MIC 0.25-1.5 µg/ml). In this study, 32.4% strains were resistant to three or more than three antibiotics and prevalence of this multi-drug resistant *S. aureus* was 45% (95%CI: 26.6-63.4) and 17.6% (95%CI: 6.7-28.5) in swine and bovine nasal samples, respectively. Four strains from pigs were borderline oxacillin-resistant *S. aureus* MIC 6-8 µg/ml, but none were *mec*A positive. Two of these strains were β-lactamase hyperproducers. Among the resistance genes *bla*Z, *tet*K, *tet*L, *tet*M, *erm*B, and *aac*A-*aph*D were found.

**Conclusion:**

Our results demonstrated the absence of *mec*A and *pv*l gene, but the presence of multi-drug resistant *S. aureus* in the nares of healthy animals which has a potential to spread in a community.

## Introduction

In food animal production, antibiotics are used extensively and are often applied sub-therapeutically for growth promotion and disease prevention in India. This has resulted in the rise of antibiotic-resistant *Staphylococcus aureus* in food animals and foods of animal origin. *S. aureus* resistance to oxacillin carrying the *mec*A gene, now known as methicillin-resistant *S. aureus* (MRSA), was first reported in the year 1961 and since then reported in hospitals, food, animals, community and the environment [1]. Recently, MRSA strains, consistent with the main livestock-associated (LA)-MRSA type have caused outbreaks in healthcare settings thereby raising the concern. In addition, a new variant of methicillin resistance determinant (*mec*C) has been described in specific LA-MRSA lineages that are primarily associated with swine, cows, and sheep but again display a broad host range [2].

Sustained efforts are therefore required to analyze antibiotic resistance (AR) patterns of *S. aureus* strains isolated from different origins, including livestock due to their changing patterns of resistance and its potential to serve as a reservoir of AR genes and MRSA. AR of *S. aureus* nasal carriage isolates from bovine and swine has not been pursued aggressively, especially in Punjab.

Therefore, the aim of this study was to figure the prevalence, phenotypic and genotypic AR pattern of *S. aureus* isolated from bovine and swine nares.

## Materials and Methods

### Ethical approvals

The study was conducted under the supervision of institutional ethical committee.

### Sampling

Nasal swabs of 47 apparently healthy bovines and 28 apparently healthy swine were obtained from the Teaching Veterinary Hospital and the pig farm of Guru Angad Dev Veterinary and Animal Sciences University (GADVASU), Ludhiana, Punjab, respectively, during February to April 2014.

### Isolation and identification of *S. aureus*

Isolation of *S. aureus* from swab samples was performed as per the bacteriological analytical manual [3]. Colonies with typical morphology on Baird-Parker agar supplemented with egg-yolk tellurite emulsion were selected and subjected to Gram-staining and catalase test. Gram and catalase positive isolates were biochemically identified as *S. aureus* using the HiStaph™ Identification Kit (HiMedia Labs, Mumbai). These isolates were further subjected to coagulase test using rabbit plasma (HiMedia Lab, Mumbai) following manufacturer’s instructions. The *S. aureus* isolates were purified and maintained in 20% (v/v) glycerol at −20°C till further processing.

### Antibiotic susceptibility testing (AST) of selected *S. aureus* strains

The AST of *S. aureus* strains was performed by the Epsilometer test. All the selected *S. aureus* strains were tested for their sensitivity to various antibiotics, *viz*., oxacillin, penicillin, tetracycline, chloramphenicol, cotrimazole, ceftriaxone, gentamicin, erythromycin, ciprofloxacin, and vancomycin using Ezy MIC™ strip (HiMedia Lab, Mumbai). AST for amoxicillin/clavulanic acid and double- disk diffusion testing (D-test) was performed by the standard disc diffusion method following CLSI guidelines [4].

### Identification of antibiotic resistant genes

*S. aureus* strains were tested for the presence of following AR genes: *bla*Z, *mec*A, *aac*A*-aph*D, *erm* (*erm*A, *erm*B, *erm*C), *tet* (efflux genes *tet*K and *tet*L, *tet*M and *tet*O of the ribosomal protection family) and *van*A, encoding for penicillin, oxacillin, gentamicin, erythromycin, tetracycline and vancomycin resistance, respectively, by amplification of the existing gene using multiplex polymerase chain reaction (PCR). Primers for the respective genes are listed in Table-1 [5-10].

**Table-1:** Primers used for detection of antibiotic resistant genes in *S. aureus*.

Gene	Oligonucleotide sequence (5`-3`)	Amplicon size	References
*aacA-aphD*	TAA TCC AAG AGC AAT AAG GGC GCC ACA CTA TCA TAA CCA CTA	227	[5]
*erm*A	AAG CGG TAA ACC CCT CTG A TTC GCA AAT CCC TTC TCA AC	190	[5]
*erm*B	CTATCTGATTGTTGAAGAAGGATT GTTTACTCTTGGTTTAGGATGAAA	142	[6]
*erm*C	AAT CGT CAA TTC CTG CAT GT TAA TCG TGG AAT ACG GGT TTG	299	[5]
*tetK*	GTA GCG ACA ATA GGT AAT AGT GTA GTG ACA ATA AAC CTC CTA	360	[5]
*tetM*	AGT GGA GCG ATT ACA GAA CAT ATG TCC TGG CGT GTC TA	158	[5]
*tetL*	GTCGTTGCGCGCTATATTCC GTGAACGGTAGCCCACCTAA	696	[7]
*tet*O	AATGAAGATTCCGACAATTT CTCATGCGTTGTAGTATTCCA	781	[7]
*mecA*	AAA ATC GAT GGT AAA GGT TGG C AGT TCT GCA GTA CCG GAT TTG C	532	[5]
*blaZ*	ACT TCA ACA CCT GCT GCT TTC TGA CCA CTT TTA TCA GCA ACC	173	[6]
*van*A	ATGAATAGAATAAAAGTTGC TCACCCCTTTAACGCTAATA	1032	[8]
*16SrDNA* (Staphylococcus genus specific)	CAG CTC GTG TCG TGA GAT GT AAT CAT TTG TCC CAC CTT CG	420	[5]
*Coa*	ATA GAG ATG CTG GTA CAG G GCT TCC GAT TGT TCG ATG C	547 (550-875)	[9]
*Nuc* (Species specific)	GCGATTGATGGTGATACGGTT AGCCAAGCCTTGACGAACTAAAGC	279	[10]

S. aureus=Staphylococcus aureus

Antibiotic resistant genes from strains were amplified, purified and sequenced (Invitrogen) for use as a control. Sequence comparisons were performed using the BLAST online program (http://www.ncbi.nlm.- nih.gov/BLAST/), and respective sequences were submitted to NCBI. *S. aureus* strain ATCC 33591 and ATCC 33592 were used as MRSA (*mec*A +ve) and MSSA (*mec*A −ve) positive control, respectively. KU872013, KP834338/KP834339, KP658721, KP658723, KP886833, KT454736, KT454737, *S. aureus* strains were used as positive control for genes *bla*Z, *aac*A-*aph*D, *tet*K, *tet*L, *tet*M, *erm*B, *erm*C, respectively.

The isolation of genomic DNA from *S. aureus* strains was done using HiPurA™ bacterial genomic DNA purification kit (HiMedia Lab, Mumbai). Each strain was subjected to a separate multiplex PCR assays for a detection of each group (gp) of genes: gp1 (*16S rDNA*-genus specific, *nuc*-species specific, *mec*A), gp2 (*tet*K, *tet*L, *tet*M and *tet*O), gp3 (*erm*A, *erm*B, *erm*C and *aac*A*-aph*D), and gp4 (*coa* and *blaZ*). Separate PCR was run for *van*A and *pvl* gene.

The PCR amplification was carried out in a total reaction volume of 25 µl containing 0.4 mM deoxynucleotide triphosphates, 4 mM MgCl_2_ (Promega, USA), 10 pmol/µl of each primer set containing forward and reverse primers, 1 U *Taq* DNA polymerase (Promega, USA), 0.01-0.2 µg template and sterilized nuclease free water was added to make up the reaction volume of 25 µl.

The cycling conditions of multiplex PCR for gp1, gp2, and gp3 and of single PCR for *van*A gene were as per methodology of Strommenger *et al*. [5] (with little modifications) and Saha *et al*. [8], respectively. However, cycling condition for *coa* and *blaZ* (gp 4) included an initial denaturation of DNA at 94°C for 45 s, followed by 30 cycles of denaturation at 94°C 20 s, annealing at 55°C for 15 s and extension at 70°C for 15 s, followed by a final extension of 2 min at 72°C and hold at 4°C. The cycling condition for *pvl* includes 94°C for 1 min, 30 cycles of denaturation at 94°C for 30 s, annealing at 55°C for 30 s, and extension at 72°C for 1 min followed by a final extension at 72°C for 5 min.

All the PCR amplicons were visualized using an ultraviolet light box after electrophoresis on a 1.5% agarose gel containing 0.5 µg/ml ethidium bromide.

### Statistical analysis

Microsoft excel was used for statistical analysis. The variables were compared using a Chi-squared or Fisher’s exact test, as appropriate. Differences were considered significant when the p<0.05. Dendrogram based on antibiogram was composed using the unweighted paired group method with Bionumeric software V07.

## Results

**Figure-1:**
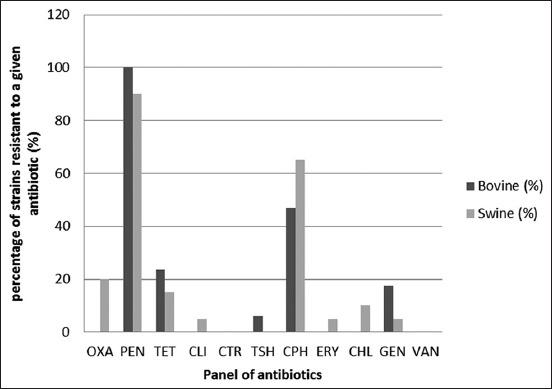
Percentage of *Staphylococcus aureus* strains resistant to different antibiotics.

The nasal cavities of 17 out of 47 randomly selected bovine and 20 out of 28 randomly selected swine were positive for *S. aureus*, representing the prevalence of 36.2% (95% confidence interval [CI]: 22.5-49.9) and 71.4% (95% CI: 54.7-88.1), respectively. Furthermore, all the *S. aureus* strains were coagulase positive except one swine nasal swab (1S1). One strain from each sample was screened against antibiotics that are of clinical importance and percentage of strains classified as susceptible, intermediate or resistant were used to summarize resistance percentage overall and disaggregated by species type (Figure-1).

## Phenotypic resistance profile

**Figure-2:**
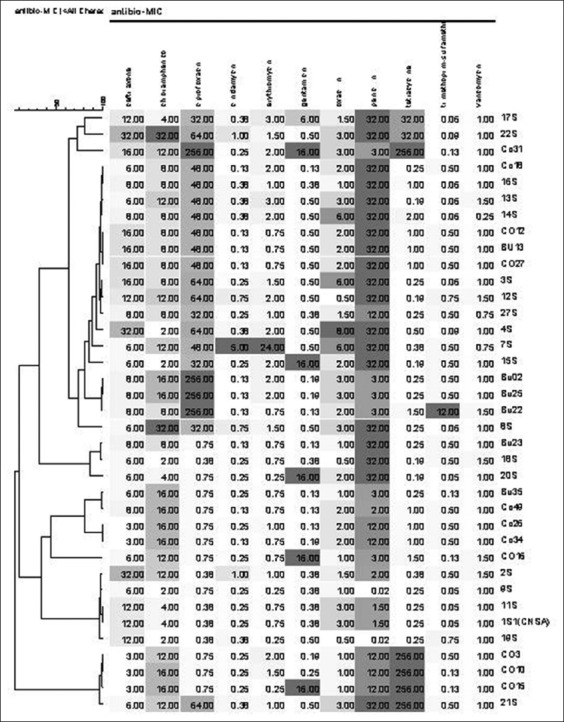
Dendrogram based on minimal inhibitory concentration antibiogram of 37 *Staphylococcus aureus* strains. The dendrogram was obtained using the unweighted paired group method with bionumeric software V07. The color indicates the susceptibility gradient with Dark-resistant, gray-intermediate resistant and white or light gray-susceptible. The top left horizontal scale indicates the number of antibiotics 1-11.

Most of the *S. aureus* strains showed higher resistance to penicillin (94.6%, MIC ≥1.5 µg/ml) followed by ciprofloxacin (56.7%, MIC ≥32 µg/ml) and tetracycline (18.9%, MIC ≥32 µg/ml). About 10-15% of the strains were resistant to gentamicin (MIC 16 µg/ml) and oxacillin (MIC 6-8 µg/ml). However, 21.6% of *S. aureus* strains showed intermediate resistance to chloramphenicol (MIC 16 µg/ml). Similarly, for erythromycin and ceftriaxone, most of the strains were intermediate resistant with MIC 1-3 µg/ml, 54.05% and MIC 16-32 µg/ml, 21.6%, respectively. Vancomycin MIC values for *S. aureus* strains were in the susceptible range (0.25-1.5 µg/ml). Around 16 unique resistance profiles (taking five antibiotics as cutoff value) were found (Figure-2).

In this study, 32.4% strains were resistant to three or more than three antibiotics, and they were designated as multidrug resistance (MDR). The prevalence of MDR *S. aureus* was 45% (95% CI: 26.6-63.4) and 17.6% (95% CI: 6.7-28.5) in swine and bovine nasal samples, respectively.

## Relationship between AR phenotype and genotype

### Penicillin

One *S. aureus* strain (Bu35) was negative for *bla*Z gene, but resistant to penicillin with MIC 3 µg/ml and was intermediately resistant to chloramphenicol (Figure-2 and Table-2).

**Table-2:** Correlation between the phenotypic and genotypic AR to penicillin, oxacillin and gentamicin in *S. aureus*.

Gene	Number of strains	Number of strains tested by E strip (MIC µg/ml)		

		Sensitive	Intermediate	Resistant
*bla*Z		Penicillin MIC≤0.12	Penicillin MIC 0.12-0.25	Penicillin MIC≥0.25
Positive	34	0	0	34
Negative	3	2	1
*mec*A		Oxacillin MIC≤2	Oxacillin MIC 2-4	Oxacillin MIC≥4
Positive	0	0	0	0
Negative	37	33	0	4
*aac*A-*aph*D		Gentamicin MIC≤4	Gentamicin MIC 8	Gentamicin MIC≥16
Positive	15	10	0	5
Negative	22	22	0	0

*S. aureus=Staphylococcus aureus,* MIC=Minimal inhibitory concentration, AR=Antibiotic resistance

### Oxacillin

All *S. aureus* strains were *mec*A negative. However, four *S. aureus* strains that were phenotypically resistant to oxacillin but genotypically *mec*A negative showed borderline resistance with MIC ranges from 6 to 8 µg/ml (Table-2). Out of these four *S. aureus* strains, S7 and S14 were β-lactamases hyperproducers (Table-3).

**Table-3:** Characteristics of isolates resistant to oxacillin but negative for the *mec*A gene by PCR.

Source	Strains	Oxacillin MIC (µg/ml)	Ceftriaxone MIC (µg/ml)	Amoxyclave inhibition zone* (mm)	*blaZ* (PCR detection)	β-lactamase hyper producers
Swine nasal swab	S3	6	16	14	+	-
Swine nasal swab	S4	8	32	15	+	-
Swine nasal swab	S7	6	6	40	+	+
Swine nasal swab	S14	6	8	35	+	+

The diameter of the inhibition zone with amoxyclave (amoxicillin-clavulanic acid: 20 and 10 µg, respectively) if exceeded 20 mm then strains were considered β-lactamases hyperproducers. Ceftriaxone MIC≤8 µg/ml - susceptible, MIC=1632 µg/ml-intermediate resistant and MIC≥64 µg/ml. MIC=Minimal inhibitory concentration, PCR=Polymerase chain reaction

### Tetracycline

The majority of the *S. aureus* strains in this study either carried *tet*K or *tet*L whereas one strain contained both *tet*K and *tet*M gene (Table-4). *tet*L was found only among swine nasal *S. aureus* strains, whereas *tet*K was common among bovine nasal *S. aureus* strains. This is agreeing with Kadlec *et al*. [11] and Ugwu *et al*. [12]. Furthermore, among *S. aureus* strains that were carrying the gene, 63.6% showed resistance to tetracycline with higher MIC values (32->256 µg/ml).

**Table-4:** Correlation between the phenotypic and genotypic AR to tetracycline in *S. aureus*.

*tet* presence	Number of strains tested by E strip (MIC µg/ml)					

	*tet*K	*tet*L	*tet*M	Sensitive (≤4)	Intermediate (8)	Resistant (≥16)
Positive	5	6	1*	4	0	7
Negative	26			26	0	

* 1 samples (Co31) possess both tetK and tetM. MIC=Minimal inhibitory concentration, AR=Antibiotic resistance, *S. aureus=Staphylococcus aureus*

### Erythromycin

Most of *S. aureus* strains were genotypically negative but phenotypically intermediate resistant with MIC 1-3 µg/ml (Table-5). In this study, all strains considered resistant or intermediate to erythromycin *in vitro* tested for inducible clindamycin resistance (D-test). None of the strains showed inducible macrolide, lincosamides, and streptogramins Type B (MLS_B_) phenotype (Ery+/Cli−, D+), but one strain (7S) showed a constitutive MLS_B_ phenotype (Erm+/Cli+, D−) and positive for *erm*B gene.

**Table-5:** Relationship between the phenotypic and genotypic AR to erythromycin *S. aureus*.

*erm* presence	Number of strains tested by E strip (MIC µg/ml)				

	*erm*B	*erm*A/*erm*C	Sensitive (≤0.5)	Intermediate (1-4)	Resistant (≥8)
Positive	5	0	0	4	1
Negative	32		16	16	0

MIC=Minimal inhibitory concentration, AR=Antibiotic resistance, *S. aureus=Staphylococcus aureus*

### Gentamicin

All *S. aureus* strains resistant to gentamicin with MIC ≥16 µg/ml showed the presence of *aac*A*-aph*D gene, and that constitutes only 33.3% of total strains positive for this gene (Table-2).

## Discussion

Penicillin, ciprofloxacin, and tetracycline are highly used in veterinary medicine and as feed additive in India [13]. This may be a reason for the increase in resistance to these antibiotics. Though low, but intermediate resistance to ceftriaxone is also a concern because ceftriaxone (third generation of cephalosporin) used to treat animals and humans especially children [14]. These results corroborate similar studies in pig, cattle and clinical isolates [15,16] and obtain in other countries [17,18] which reflects the predominant use of these drugs in pig and dairy cattle husbandry worldwide.

There was no significant difference between the percentage of AR among the bovine and swine nasal *S. aureus* strains (Fisher’s exact test; p=0.478), but higher prevalence of MDR *S. aureus* was observed in swine as compared to bovine. Thereby, our data show that bovine and swine nares are often contaminated with MDR *S. aureus*, which is the public health concern because direct contact with livestock colonized with resistant bacteria is the most documented routes of resistance transmission from the animals into human populations [19].

There was a statistically significant relationship (p<0.05) between phenotypes and genotype resistance pattern in *S. aureus* strains subjected to the antibiotic penicillin, tetracycline, gentamicin, and erythromycin. However, not all the penicillin-resistant *S. aureus* strains exhibited genotypic resistance to penicillin (Table-2), corresponding well with the findings of the previous studies [20,21]. This may be because the phenotypic resistance has been caused by point mutations, biofilm formation or antibiotic tolerance [22]. Thereby, our results suggest that *bla*Z may play a major role but cannot be used alone as an indicator for penicillin resistance.

Borderline oxacillin-resistant *S. aureus* (BORSA) has been another relatively frequently observed phenotype among *S. aureus* strains. These strains are cefoxitin/ceftriaxone susceptible and do not carry the *mec*A or *mec*C genes, but are shown oxacillin resistance MIC between 1 and 8 µg/ml [23]. In this study, All *S. aureus* strains were negative for *mec*A gene and four of those strains were BORSA. Such pattern may be because of hyperproduction of β-lactamases, production of normal penicillin-binding protein 2 with altered binding capacity or variant of *mec*A gene (*mec*C) [24]. To exclude the possibility of hyperproduction of β-lactamase, amoxyclave antibiotic disk diffusion test was performed [6]. Two of those strains were found β-lactamases hyperproducers and other two were not (Table-3). Martineau *et al*. [6] also reported exist of isolates that were phenotypically oxacillin resistant but negative for *mec*A gene. Likewise Pereira *et al*. [25] reported 38% of *S. aureus* strains phenotypically resistant to oxacillin but only 0.68% of those strains showed the presence of *mec*A gene. In conclusion, our result showed the prevalence of BORSA that is of public health concern because the incidences of BORSA has been reported among human clinical strains [26] and have also been detected in food of animal origin, cattle and pigs [27].

Farmers and veterinarians harbored tetracycline resistant strains more frequently than people without contact with livestock especially pigs [28]. Our result reported the presence of *tet*K, *tet*L and *tet*M genes in bovine and swine nares and is of public health concern because the *tet*K and *tet*M genes are on mobile genetic element, such as small plasmids and conjugative transposons of *S. aureus* [7] that can spread and cause treatment failure both in veterinary and human medicine.

A form of acquired simultaneous resistance to MLS_B_ in clinical isolates is due to evolutionary variants of *erm* genes [11]. The strains in the current study only had *erm*B gene. This is in accordance with the study that too reported *erm*B gene in animal strains [6]. Most of the strains were intermediate resistant to erythromycin but *erm* gene negative. This intermediate resistance to erythromycin can be possibly associated with mechanism not yet characterized in staphylococcus or may be due to another gene such as *msr*A and *mph*C––corroborating with past research [6,12].

In this study, most of the strains were *aac*A*-aph*D positive but phenotypically sensitive to gentamicin (Table-2). This is the issue of concern from a clinical prospective as the susceptible strains harboring but not expressing an AR gene is considered as potentially resistant to that antibiotic because there are the experiments that strongly suggest that susceptible strains harboring a resistance gene have the potential to develop resistance on *in vivo* selection of the appropriate antimicrobial agent [6]. None of the strain was *pvl* positive.

## Conclusion

Hitherto there is no direct evidence that the food can contribute to the community-associated-MRSA but the direct contact with colonized animals, especially swine contact now appears to be a significant risk factor for colonization with MDR *S. aureus* and MRSA (LA-MRSA), thereby explains the relevance of this type of studies. To our knowledge, this is the first short study in the Punjab examines the prevalence and characteristics of MDR *S. aureus* and screening for MRSA in bovine and swine nares. Of particular concern in this study was the small sample size, but clearly, shows the requirement of further study at the farm and retail levels involving large sample size over time at different location to better assess the presence of MDR *S. aureus* and MRSA in livestock and the risk to livestock handlers and consumers. Although all strains were susceptible to vancomycin and negative for *mec*A and *pvl* gene, the *S. aureus* strains have demonstrated resistance to more than two antibiotics. Our result showed the prevalence of BORSA that is of public health concern because the incidences of BORSA have been reported in hospitals. We also observed that most strains presented the genes of resistance, although these were not being expressed, yet demonstrating the future potential for these strains to become resistant to the evaluated antimicrobial agents.

## Authors’ Contributions

The study was designed by RS and JPSG. Laboratory work was done by AS. SK and AS prepared manuscript while as RS and AS analyzed data. All authors read and approved the final manuscript.
